# Privacy Policies for Apps Targeted Toward Youth: Descriptive Analysis of Readability

**DOI:** 10.2196/mhealth.7626

**Published:** 2018-01-04

**Authors:** Gitanjali Das, Cynthia Cheung, Camille Nebeker, Matthew Bietz, Cinnamon Bloss

**Affiliations:** ^1^ Department of Family Medicine and Public Health University of California San Diego La Jolla, CA United States; ^2^ Division of Biological Sciences University of California San Diego La Jolla, CA United States; ^3^ Qualcomm Institute California Institute for Telecommunications and Technology University of California San Diego La Jolla, CA United States; ^4^ Donald Bren School of Information and Computer Sciences Department of Informatics University of California Irvine Irvine, CA United States; ^5^ Department of Psychiatry University of California San Diego La Jolla, CA United States

**Keywords:** privacy, comprehension, mobile applications, adolescent

## Abstract

**Background:**

Due to the growing availability of consumer information, the protection of personal data is of increasing concern.

**Objective:**

We assessed readability metrics of privacy policies for apps that are either available to or targeted toward youth to inform strategies to educate and protect youth from unintentional sharing of personal data.

**Methods:**

We reviewed the 1200 highest ranked apps from the Apple and Google Play Stores and systematically selected apps geared toward youth. After applying exclusion criteria, 99 highly ranked apps geared toward minors remained, 64 of which had a privacy policy. We obtained and analyzed these privacy policies using reading grade level (RGL) as a metric. Policies were further compared as a function of app category (free vs paid; entertainment vs social networking vs utility).

**Results:**

Analysis of privacy policies for these 64 apps revealed an average RGL of 12.78, which is well above the average reading level (8.0) of adults in the United States. There was also a small but statistically significant difference in word count as a function of app category (entertainment: 2546 words, social networking: 3493 words, and utility: 1038 words; *P*=.02).

**Conclusions:**

Although users must agree to privacy policies to access digital tools and products, readability analyses suggest that these agreements are not comprehensible to most adults, let alone youth. We propose that stakeholders, including pediatricians and other health care professionals, play a role in educating youth and their guardians about the use of Web-based services and potential privacy risks, including the unintentional sharing of personal data.

## Introduction

Both Apple and Android have recently surpassed 1.5 million apps available on their respective markets [1]. Most of these apps collect user statistics and are able to make use of the built-in sensors on one’s mobile phone to track movement, location, and other personal behavior and activity [2]. Although the use of built-in sensors may simplify the user interface and improve user experience, it can also allow app developers and third parties to gather potentially sensitive information about the consumer [2]. Due to the growing availability of consumer information, protection of personal data is of increasing concern.

Privacy policies should inform users of the risks of the product they are about to use. Whereas most users may not read the privacy policy, if they have concerns about their privacy while using an app, they should be able to refer back to the policy to understand how their information is being collected or used. Although the Federal Trade Commission (FTC) recommends that mobile apps make privacy statements available to app users [3], not all apps have privacy policies. Furthermore, there are no clear standards regarding the accessibility of privacy statements for the average consumer, so privacy policies are often lengthy and difficult to read and comprehend [4]. In fact, an analysis of the privacy policies of mobile health apps conducted in 2015 found that most mobile health apps did not have privacy policies. Of the privacy policies that did exist, two-thirds of them did not focus solely on the app itself but instead addressed several apps or services offered by the developer. The available policies also did not make privacy practices transparent to the readers and had a high reading grade level (RGL) [4]. This presents a unique set of challenges when considering apps targeted toward minors.

Two existing regulations have attempted to address these issues: the FTC’s Children’s Online Privacy Protection Act (COPPA) and the California Online Privacy Protection Act (CalOPPA). The COPPA took effect in 2000 and created stipulations for the collection, usage, and sharing of information from children under 13 years by Web-based services. In 2013, COPPA rules were updated to address the privacy threats associated with “big data” and the ability for mobile apps and websites to collect highly granular information from consumers such as geolocation, relationships with friends, and different behaviors and preferences. The new COPPA guidelines also addressed parental concerns about websites collecting information about location, friends and contacts, and tracking software associated with mobile apps [5]. Similarly, the CalOPPA imposed regulations on apps available to California residents, requiring them to have a privacy statement informing consumers how their information is collected and shared [6]. CalOPPA also requires privacy statements to include a list of personally identifiable information being collected and a list of third parties with whom information is shared [6]. Unfortunately, it is still often unclear how third parties are collecting information that is entered into the app [7]. This calls into question the effectiveness of such a policy if users are not aware that apps are collecting their information.

The unnoticed involvement of third parties is of particular concern when considering apps targeted toward minors. Although the COPPA legally restricts the ways in which information from minors younger than 13 years can be collected and used, language in the COPPA excludes teenagers from 13 to 18 years of age from these same protections. Although the responsibility of monitoring a child’s Web safety has traditionally fallen on the child’s parents [8], the teenage years are a time when parents tend to have less direct oversight of Web-based activities. Teens who use mobile apps and websites are less likely to involve their parents when interfacing with and providing information to Web-based services [9] and may not be fully aware of how their information is collected and used. An open question, then, is the extent to which parents are able to adequately understand and advise on the privacy implications of their children’s Web-based activities.

Internet safety has become a public health issue that concerns health care providers. The American Academy of Pediatrics (AAP) encourages parents to open a dialogue with their children about Web safety [10]. However, the lack of parental involvement in Web-based activity potentially leaves teens in a vulnerable situation regarding personal privacy and Web-based behaviors. For example, location tracking is a known safety concern particularly for teenage girls [9], making it important for teens to be aware of location-tracking features on the apps they download. Additionally, the increasing prevalence of social networking features in popular apps can expose youth to cyberbullying or unsuitable material, which can lead to long-term mental health consequences [11]. Although some research has shown that teenagers will take steps to protect their privacy by avoiding apps or disabling features that track their movements or usage [9], it is unclear whether the majority of teens are actually aware of the need to take such measures. Given that an estimated one in 3 Internet users is younger than 18 years [12], the implications of this issue are considerable. A 2012 analysis of app permissions and risk signals concluded that popular apps require more permissions for greater functionality, yet there are no reliable “risk signals” that alert users to the privacy risks associated with the app [13]. Privacy policies, such as an informed consent document, should be written in a way for users to understand their privacy risk when using an app. This study was designed to evaluate the readability of privacy policies for apps that are available to and targeted toward youth. Our goal was to inform strategies to educate and protect youth from unintentional sharing of personal data. The overarching privacy principles state that patients must be able to easily find and read the privacy policy of their health technology, and they have the right to refuse participation. The readability statistics collected in this study are compared with the Patient Privacy Rights’ Trust Framework (PPR TF) principle #1 criteria on ability to find and understand privacy policies, which recommends an RGL of 12.0 or lower and a Flesch reading ease of 45.0 [14].

## Methods

### App Selection Process

**Figure 1:**
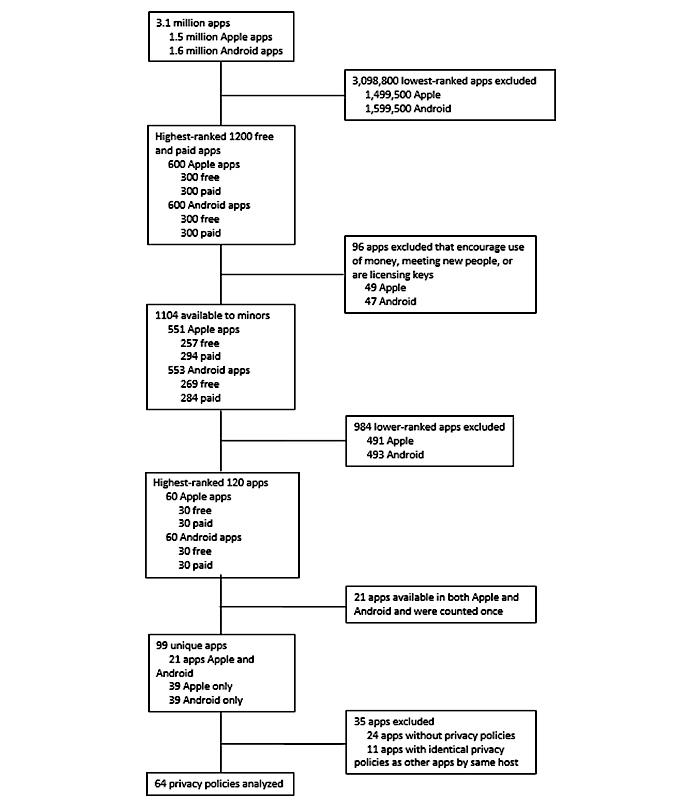
App selection process flowchart (completed March 2016).

Figure 1 outlines the app selection process used. The Apple App Store and the Google Play Store have a combined total of over 3 million apps available for download on mobile devices [15,16]. Each store ranks their apps according to their respective ranking formulas, which take into account app ratings, reviews, and number of downloads. We identified and analyzed the highest-ranked 300 free and 300 paid apps in the Apple App Store and the highest-ranked 300 free and 300 paid apps in the Google Play Store, for a total of 1200 apps, which were reviewed manually.

#### Focus on Youth

We made efforts to focus our study on apps actually used by youth, and this was done by further narrowing down the selection from the initial 1200 apps identified. Apps were characterized as available to and targeted toward minors if they generally did not require the use of money and did not facilitate interaction with unknown people. Specific exclusion criteria included apps that (1) encourage the use of money outside in-app purchases (eg, shopping, travel, or real-estate apps), (2) facilitate interaction with unknown people (eg, dating or ride-service apps), (3) are focused on tracking pregnancies or newborn development, or (4) serve as licensing keys that unlock premium features of other apps (only in the Google Play Store). Shopping apps included apps related to specific stores or corporations (eg, Kohl’s, Walmart, or Amazon), buy and sell apps (eg, letgo or eBay), and coupon or discount apps (eg, Groupon). Shopping apps did not include subscription streaming services such as HBO Now or Netflix. Dating and ride-service apps, including Tinder and Uber, were omitted because interaction with strangers is discouraged for youth.

Pregnancy and newborn development tracking apps were omitted because having and raising children is less common among teenagers and youth. A total of 96 apps were omitted. All other apps were included.

#### Reliability

To determine the reliability of the exclusion criteria, a second rater who had not seen the original list of 1200 apps applied the exclusion criteria to a random sample of 120 apps (30 per app type—Apple Free, Apple Paid, Google Play Free, and Google Play Paid). Out of the 120 apps, there was disagreement on only one app, yielding a kappa statistic of .94 (*P*<.001), which demonstrates high interrater agreement [17]. After discussion, the 2 raters came to consensus on the one app of disagreement and included it in the sample as “available to youth.”

For the analysis of the apps, in each of the four app types, the highest ranked 30 apps, representing 10.00% (120/1200) of the apps, were reviewed for availability of a privacy policy. A total of 120 apps were considered a feasible number of privacy policies to analyze using a readability calculator. Of these 120 apps, 21 were available in both the Apple and Google stores and were analyzed only once. Out of the final 99 apps, 24 apps did not have privacy policies, and 11 apps had identical privacy policies because of those apps being products of the same developer. This left a total of 64 unique documents for our final readability analysis. Privacy policies of apps were found either via direct link to the privacy policy from the respective app store or from a link to the website of the app developer.

#### Readability Analysis

Comprehensibility was measured as “readability,” or the ease of understanding the given text. Readability was used as a measure of comprehensibility, as it provides an unbiased numerical value reflective of comprehensibility. Readability statistics of privacy policies for apps from the Apple and Google Play app stores were calculated using a Web-based readability calculator and analyzed. The average RGL was then compared with the average RGL of adults in the United States. Notably, there are no standards or guidelines for the readability of mobile app privacy policies, so the readability statistics were also compared with the PPR TF. The PPR TF is a set of criteria that measure how technology affects patient privacy. These criteria were developed by the Coalition for Patient Privacy, in collaboration with others, to offer suggested standards on how patient privacy can be protected.

The 64 privacy policies were entered into a Web-based readability calculator, the Readability Test Tool (WebpageFX, Inc, Harrisburg, PA) [18], which is one of multiple free resources that calculate readability. Before selecting this tool, privacy policies were entered into multiple Web-based calculators. As most tools were found to produce fairly consistent results, the Readability Test Tool was used because of its simple user interface.

Statistics collected from the readability calculator were word count, Flesch reading ease, Flesch-Kincaid RGL, Gunning-Fog RGL, simplified measure of Gobbledygook (SMOG) RGL, sentence count, and number of complex words. Flesch reading ease computes a score on a scale from 0 to 100 with higher numbers representing greater reading ease. Flesch-Kincaid, Gunning-Fog RGL, and SMOG RGL are calculated by taking into account the sentence length and average word length. Gunning-Fog uses the average word length to determine the percentage of complex words or words with greater than three syllables. SMOG RGL typically overestimates the RGL of the text, and Flesh-Kincaid typically underestimates RGL. For a more accurate metric, RGL was calculated as the average of Flesch-Kincaid RGL, Gunning-Fog RGL, and SMOG RGL (Table 1).

### Data Analysis

Mean RGL of the 64 apps was compared with the average adult reading level in the United States and to the PPR TF recommended RGL of 12.0. The Flesch reading ease score was compared with the PPR TF recommended reading ease score of 45.0. Apps were also divided into three broad app categories (entertainment, social networking, and utility) based on app store classifications. Entertainment apps included games, music, and video apps (eg, Angry Birds, Spotify, and Netflix). Social networking apps were categorized as such by the app stores and included messaging services associated with social networking (eg, Snapchat, Facebook Messenger, and Instagram). Utility apps encompassed all apps for general use and apps that did not fit into the other two categories (eg, flashlight, word processing, or email apps). RGL of the three categories were compared using a one-way analysis of variance (ANOVA). All reported *P* values are uncorrected.

**Table 1:** Flesch-Kincaid, Gunning-Fog, simplified measure of Gobbledygook (SMOG), and average reading grade levels (RGLs) for all apps included in the analysis. The average reading level column is the average of Flesch-Kincaid, Gunning-Fog, and SMOG RGLs.

App name	Average reading level	Flesch-Kincaid reading level	Gunning-Fog	SMOG^a^
Disney Build It: Frozen	17.1	16.8	19.4	15
Subway surfers	15.9	16.1	18.2	13.6
Nova Launcher Prime	15.6	15.8	16.9	14.1
Monument Valley	15.5	15.9	16.6	14
WhatsApp	15.5	16.2	17.4	13
Du Battery Saver and phone charger	15.2	14.5	17.9	13.3
Netflix	14.9	14.6	17.2	13
Grand Theft Auto: San Andreas	14.7	14.5	16.4	13.1
Mobile Strike	14.6	14.6	16.6	12.5
Pages	14.2	13.8	16.2	12.6
Terraria	14.2	13.7	16.3	12.5
Faily brakes	14.1	13.8	16.1	12.3
Pandora	14.1	13.9	16	12.5
Rolling Sky	14	13.4	16.4	12.3
Stick Texting: The Emoji Killer	13.9	13.8	15.7	12.4
Gmail	13.8	13.5	15.9	11.9
Assassin’s Creed Identity	13.7	13.8	14.8	12.5
Minecraft: Story Mode	13.7	13.5	15.6	12
Angry Birds	13.6	13.3	15.2	12.4
NBA 2K16	13.5	13.3	15	12.2
Candy Crush Jelly Saga	13.4	13	15	12.2
FaceSwap Live Lite	13.4	13.3	14.5	12.3
Ultimate Guitar Tabs and Chords	13.3	13	14.8	12
Twitter	13.2	13.2	15.2	11.2
Agar.io	12.9	12.3	15.3	11.3
Hitman: Sniper	12.9	12.7	14.2	11.7
Kimoji	12.9	12.6	14.7	11.5
Spotify Music	12.8	12.5	14.3	11.7
VivaVideo Pro	12.8	12.3	14.8	11.2
Facetune	12.7	12.7	13.9	11.6
Heads Up	12.7	12.3	14.5	11.3
Swype keyboard	12.7	12.5	13.9	11.6
Fishdom: Deep Dive	12.6	12.4	14.4	11.1
Game of Life Classic Edition	12.6	12.4	14.3	11.2
Geometry Dash	12.6	12.2	14.4	11.3
Power Clean: Optimize cleaner	12.6	12.4	13.9	11.5
Snapchat	12.5	12.2	14.6	10.8
Super Bright LED Flashlight	12.5	12.3	13.5	11.8
Clash Royale	12.4	11.8	14.4	11.1
Plague Inc	12.4	12.6	13.1	11.6
Sleep Cycle Alarm Clock	12.4	12.4	13.2	11.6
Bloon TD 5	12.3	12.3	14.1	10.6
Facebook	12.3	11.8	14.3	10.8
Instagram	12.3	12.1	13.9	11
Akinator the Genie	12.2	12.3	12.8	11.4
YouTube	12.2	11.7	14.4	10.6
Please Don’t Touch Anything	11.9	11.6	12.8	11.2
Musical.ly	11.8	11.5	12.7	11.2
ZEDGE	11.8	11.4	13.4	10.7
Kik	11.7	11.3	13.3	10.6
PianoTiles 2	11.6	11.4	12.6	10.9
Dragon Land	11.4	10.7	12.8	10.6
Kika Emoji Keyboard	11.3	11	12.9	10
NeoMonsters	11.3	10.8	12.8	10.3
Pinterest	11.3	10.8	12.9	10.2
Toca Lab	11.2	11	12.6	10
Afterlight	10.8	10.4	11.9	10.2
Minecraft pocket edition	10.7	10.2	11.9	10.1
Badland 2	10.5	9.7	12.3	9.4
True Skate	10.2	10	11.4	9.1
Pocket Casts	10	9.2	11.5	9.3
SuperPhoto Full	10	9.4	11.7	9
The Room Three	10	9.3	11.7	9.1
Papa’s Freezeria To Go	8.5	8.6	8.8	8.2

^a^SMOG: simplified measure of Gobbledygook.

## Results

### Readability

**Figure 2:**
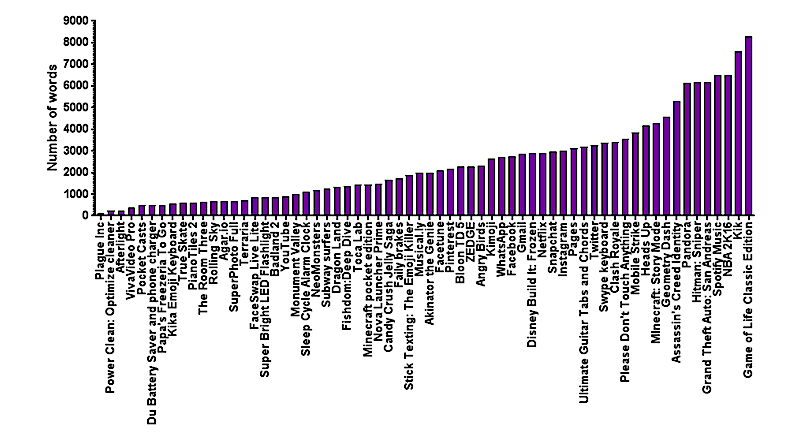
Privacy policy word count (N=64 apps). The average word count of the privacy policies was 2425 words. The Game of Life Classic Edition had the highest word count at 8290 words, and Plague Inc had the lowest word count at 140 words.

**Figure 3:**
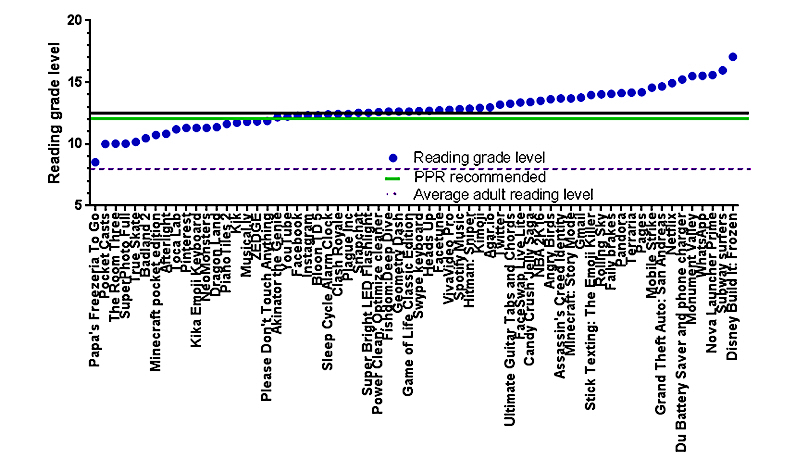
Privacy policy reading grade level (RGL; N=64). The RGL was an average of the Flesch-Kincaid, Gunning Fog, and simplified measure of Gobbledygook (SMOG) RGLs. The mean RGL of all the apps was 12.78, which is equivalent to a freshman in college. This average level is also higher than the Patient Privacy Rights (PPR) recommended RGL of 12.00 and higher than the US average adult RGL of 8.00. In terms of the individual apps, the highest RGL was for Disney Build It: Frozen at 17.07, which is equivalent to a graduate student reading level. The lowest RGL was for Papa’s Freezeria To Go at 8.53.

The privacy policies reviewed in our analysis had a mean length of 2425 words (standard deviation [SD] 1965) and ranged from 140 to 8290 words (Tables 2 and 3 and Figure 2). Privacy policies had a mean RGL of 12.78 (SD 1.611; Tables 2 and 3 and Figure 3). The correlation between privacy policy length and RGL was not statistically significant (*r*=.2452, *P*>.05, N=64). The mean Flesch reading ease was 42.73 (SD 6.991).

### Policy Readability Versus Recommended Standards

Importantly, none of the discovered privacy policies had an RGL below the average adult RGL in the United States of 8.0 (Figure 3). Privacy policies also had an average Flesch reading ease of 42.73 (SD 6.991), which is lower (ie, less readable) than the 45.0 recommended reading ease by the PPR (*P*<.05; Figure 3). The average RGL of 12.78 is similar to the PPR TF recommended RGL of 12.0.

### App Category Comparisons

The readability of policies from 30 free apps and 34 paid apps were compared. Free apps had an average RGL of 13.09 (SD 1.304), and paid apps had an average RGL of 12.51 (SD 1.815). Data are shown in Table 2 and illustrate no significant differences between free and paid apps on any of the metrics examined (*P*>.05). Apps were also divided into three broad categories (entertainment, social networking, and utility), as previously described. When privacy policies from these apps were compared as a function of category, we observed a significant difference in word count between the categories (Table 3), with social networking having the highest word count and utility the lowest. There were, however, no significant differences in average RGL.

**Table 2:** Mean readability statistics. Free versus paid: comparison of mean reading grade level (RGL), mean word count, and mean reading ease between free and paid apps from both the Android and Apple markets. The *P* values for the *t* tests between the two app types show that there is no significant difference between mean RGL, word count, and reading ease.

Statistic	All apps^a^	Free apps	Paid apps	*P* value
N	64	30	34	--
Mean RGL^b^	12.78	13.09	12.51	.15
Mean word count	2425	2355	2487	.79
Mean Flesch reading ease	42.73	42.3	43.1	.65

^a^Column summarizes results for all apps included in the analysis; it was not included in the significance test for the *P* value in the last column.

^b^RGL: reading grade level.

**Table 3:** Mean readability statistics. Entertainment versus social networking versus utility: comparison of mean reading grade level (RGL), mean word count, and mean reading ease between entertainment, social networking, and utility apps. The *P* values for the analysis of variance (ANOVA) tests show that there is no significant difference in the mean RGL and reading ease between the app categories, but there is a significant difference in mean word count.

Statistic	All apps^a^	Entertainment	Social networking	Utility	*P* value
N	64	44	7	13	--
Mean RGL^b^	12.78	12.84	12.7	12.62	.93
Mean word count	2425	2546	3493	1038	.02
Mean Flesch reading ease	42.73	42	46.46	43.37	.31

^a^Column summarizes results for all apps included in the analysis; it was not included in the significance test for the *P* value in the last column.

^b^RGL: reading grade level.

## Discussion

### Principal Findings

Analysis of privacy policies for 64 popular apps targeted toward youth revealed an average reading level of 12.78 or the equivalent RGL of a first year college student. Although this RGL is similar to the reading level recommended by the PPR TF, it is well above the average reading level of adults in the United States. These findings are similar to those from a 2015 study (Sunyaev et al), which noted that app developers and companies are not transparent about their privacy practices through their privacy policies [4]. Although users must agree to app privacy policies to access digital tools and products, these agreements are not comprehensible by the average adult, let alone youth. Because companies often collect, use, and sell users’ personal information, it is concerning that agreements describing and governing these activities are not accessible to most users. We propose that stakeholders, including pediatricians and other health care professionals, could play a role in educating youth and their guardians about the use of Web-based services and potential privacy risks, including the unintentional sharing of personal data. However, considering the complexities of privacy policy agreements, there may be a need for further tools and training to help such stakeholders, including health care workers, understand, navigate, and educate others about Web-based privacy and Internet safety.

Most parents are concerned about their child’s safety on the Internet. Whereas many have taken steps to protect their child’s safety while using the Web, such as through discussions with their children, it is often difficult for parents to know how their child’s privacy is protected on the Internet [19]. About 40% of parents of Internet users have read the privacy policies of the apps that their children are using. Previous studies that have assessed privacy policies of mobile apps have concluded that college-level literacy is required to comprehend the text of privacy statements [20]. Likewise, our study reached similar conclusions even though the apps selected for analysis were specifically directed toward children and teenagers. Apps that are available to teenagers should have privacy statements that teenagers can understand, and apps that are available to children should have privacy statements that are accessible by their parents or guardians. To be COPPA compliant, apps and websites should post a policy regarding their privacy practices so that parents are aware of how information is collected and used, and these policies must be readable and comprehensible [21].

Results from a 2013 study conducted by the Pew Research Center show that 70% of teen Internet users do seek out advice about their Internet safety. Many teenagers turn to friends, peers, or their parents for advice about privacy settings on Web applications. The results of the Pew study also show that teenagers of all racial and socioeconomic backgrounds seek advice about Internet safety, but white teenagers are more likely than black or Hispanic teenagers to talk to their parents about Web privacy. Youth should have a trusted adult they can consult when considering privacy expectations with their Web presence. By having privacy policies written so that youth can understand them, children and teenagers are afforded a sense of autonomy over their Internet practices. They will be able to make informed decisions about what kind of privacy settings they desire on their Web-based accounts, and they can discuss these privacy settings and their safety with a trusted adult [9].

Much of the inaccessible language in privacy policies stems from legal terminology used by corporations to protect themselves from potential liability. We identified excerpts from privacy policies in our study with the highest RGL (Table 4). Use of terms such as “cookies” and “third-party site” may contribute to comprehension difficulties, as well as complex phrases that use other jargon not in common parlance. It is well known that many users do not read privacy statements when they do download an app, and one possible reason for this may be the fact that they are difficult to comprehend. A potential solution is to require app developers to have versions of their privacy statements that translate the legal terminology in a way that is easy to understand. For example, Twitter’s privacy policy includes one sentence “tips” that summarize different sections of the policy [22]. These tips are short and easy to read and allow users to better understand how their personal information is being used.

We noted that even the PPR TF criteria that was used as a base of comparison for readability in this study has recommended standards that are too difficult for the average adult in the United States to comprehend, as they recommend a RGL of 12.0. We recommend that a new set of guidelines for privacy policies target the average adult in the United States, with an average RGL of 8.0 or lower, a Flesch reading ease score of 70 or higher, and a word count of less than 500 words. These standards would also be understood by most high school students, allowing teenagers to read and comprehend privacy policies for the apps they download and potentially gain a better understanding of how their personal data are collected, used, and potentially sold to third parties.

The complexity and thus incomprehensibility of privacy policies poses a serious Internet safety concern for the youth in particular. A recent study on digital monitoring activity among teenagers shows that most parents do talk to their teenage children about appropriate Web behavior and what they should share on the Internet; however, most parents do not have these talks as frequently as they speak to their children about offline behavior [23]. With the increasing use of Web-based applications in entertainment, education, and social networking, young people are making more and more information available over the Web, potentially leading to harmful consequences.

Introducing educational curricula in schools about Web-based safety and increasing exposure to safe Internet practices may be an avenue to explore empirically. These curricula could provide children and adolescents with the tools they need to understand privacy risks and make choices about how their personal data are stored and shared over the Internet. Such resources are particularly important for older teenagers, who are less likely than younger children to involve their parents or ask for advice about Web privacy [9]. Indeed, teenagers are often already in the position of making their own choices about their behavior and practices in Web-based and digital environments. Web-based safety programs, such as the one developed by Common Sense Education, allow teachers to tailor their curricula to specific grade levels to make Internet safety relevant to minors of different ages [24].

**Table 4:** Sample text from privacy policies with highest reading grade level (top 5).

App name	Word count	Reading grade level	Sample text from privacy policy
Disney Build It: Frozen	2880	17.07	“We collect...Usage, viewing and technical data, including your device identifier or IP address, when you visit our sites...or open emails we send.”
			“We acquire information from other trusted sources...”
Subway Surfers	1272	15.97	“We log information about your use of the App...”
			“...if you log into the App using a third-party site or platform such as Facebook, we may access information about you from that site or platform...”
			“We may allow third parties to serve contextual advertisements and provide analytics services in connection with the App. These entities may use various identifiers to collect information...”
Nova Launcher Prime	1487	15.60	“Information collected automatically from this Application (or third party services employed in this Application), which can include: the IP addresses or domain names of the computers utilized by the Users who use this Application...the country of origin...”
WhatsApp	2701	15.53	“WhatsApp will periodically access your address book or contact list on your mobile phone...”
			“WhatsApp uses both session cookies and persistent cookies. A persistent cookie remains after you close your browser...”
Monument Valley	984	15.50	“For operation and maintenance purposes, this Application and any third party services may collect files that record interaction with this Application (System Logs) or use for this purpose other Personal Data (such as IP Address).”
			“This Application does not support “Do Not Track” requests.”

Given the ubiquitous nature of Web-based applications and the increasing frequency of use among children and adolescents, combined with the potential for harm if these are used inappropriately, health care providers may need to consider how to address these harms in the context of their overall care of underage patients. Using clinicians as a vehicle for counseling patients on privacy and app safety practices would be analogous to the ways in which health professionals play an important role in informing patients about practices to promote a healthy lifestyle (eg, physical activity and nutrition). For example, health care providers who interact with youth (eg, orthodontists, dentists, or pediatricians) can leverage their access to youth to share information about safety practices to enhance protection of youth in Web-based settings. However, to do that, a systematic approach to document the need for and, subsequently, appropriate guidelines directed to the clinician, would be needed.

### Conclusions

Overall, Internet safety has increasingly become a public health issue. Whereas parents may have the primary responsibility for Internet safety education [8], the literature documents research findings that underscore the expertise required to understand privacy policies. The AAP has posted a guide on their website to assist parents in opening a dialogue to talk to their kids about Internet safety and social media [10]. Social networking features have become increasingly prevalent in apps—even apps that are not directly associated with social media are often linked to Facebook accounts or have the option to share on social networking. This expansive network increases opportunities for exposure to cyberbullying or material that is unsuitable for minors, which can lead to mental health and safety issues in the pediatric population [11]. Until there are clear standards for pediatricians and other health care providers specific to privacy and app safety education, they can assist by sharing information about available tools and educational resources.

Finally, institutional resources should be developed to help health professionals fulfill this role. An example of this is the AAP policy statement “Media Use in School-Aged Children and Adolescents” [25] that specifically highlights the privacy risks of social media and other Web-based activities and recognizes pediatricians’ role in helping parents set rules for Web-based activities and mentor their children about Web safety. Although the AAP tools are a good beginning, there is a need for further tools and training to help health care workers understand, navigate, and educate others about Web-based privacy and Internet safety. Overall, there is evidence that youth are concerned about maintaining their privacy, so training pediatricians and other health care providers to address privacy concerns with their patients will provide an additional safe place to ask questions and open a dialogue about Internet safety.

## Abbreviations

AAP: American Academy of Pediatrics
ANOVA: analysis of variance
COPPA: Children’s Online Privacy Protection Act
CalOPPA: California Online Privacy Protection Act
FTC: Federal Trade Commission
PPR TF: Patient Privacy Rights’ Trust Framework
RGL: reading grade level
SD: standard deviation
SMOG: simplified measure of Gobbledygook
